# DNA Methylation of PI3K/AKT Pathway-Related Genes Predicts Outcome in Patients with Pancreatic Cancer: A Comprehensive Bioinformatics-Based Study

**DOI:** 10.3390/cancers13246354

**Published:** 2021-12-17

**Authors:** Inês Faleiro, Vânia Palma Roberto, Secil Demirkol Canli, Nicolas A. Fraunhoffer, Juan Iovanna, Ali Osmay Gure, Wolfgang Link, Pedro Castelo-Branco

**Affiliations:** 1 Faculty of Medicine and Biomedical Sciences (FMCB), Campus de Gambelas, University of Algarve, 8005-139 Faro, Portugal; i.faleiro@campus.fct.unl.pt; 2 Algarve Biomedical Center Research Institute (ABC-RI), 8005-139 Faro, Portugal; 3 Instituto de Medicina Molecular João Lobo Antunes (IMM), Faculty of Medicine, University of Lisbon, 1649-028 Lisbon, Portugal; 4 Champalimaud Research Program, Champalimaud Center for the Unknown, 1400-038 Lisbon, Portugal; 5 Centre of Marine Sciences (CCMAR), University of Algarve, 8005-139 Faro, Portugal; 6 Molecular Pathology Application and Research Center, Hacettepe University, 06100 Ankara, Turkey; secil.demirkol@hacettepe.edu.tr; 7 Centre de Recherche en Cancérologie de Marseille (CRCM), INSERM U1068, CNRS UMR 7258, Institut Paoli-Calmettes, Aix-Marseille Université, Parc Scientifique et Technologique de Luminy, 13288 Marseille, France; nicolasfraunhoffernavarro@gmail.com (N.A.F.); juan.iovanna@inserm.fr (J.I.); 8 Department of Medical Biology, Acibadem University, 34684 Istanbul, Turkey; ali.gure@acibadem.edu.tr; 9 Instituto de Investigaciones Biomédicas Alberto Sols (CSIC-UAM), Arturo Duperier 4, 28029 Madrid, Spain; wlink222@gmail.com

**Keywords:** pancreatic cancer, epigenetics, DNA methylation, PI3K/AKT pathway

## Abstract

**Simple Summary:**

Pancreatic cancer is a highly lethal malignancy. Dysregulation of epigenetic mechanisms leads to abnormal patterns of gene expression contributing to the development and progression of cancer. We explored the ability of DNA methylation of PI3K-related genes to differentiate between malignant and healthy pancreatic tissue using distinct pancreatic cancer cohorts, and found that the methylation levels of the *ITGA4*, *SFN*, *ITGA2*, and *PIK3R1* genes are altered in tumour samples since the early stages of malignant transformation and could serve as new diagnostic tools. We also demonstrate that these alterations correlate with overall survival and recurrence-free survival of the patients suggesting that its assessment can serve as independent prognostic indicators of patients’ survival with higher sensitivity and specificity than the currently implemented biomarkers. Therefore, the methylation profile of genes involved in this pathway may be an alternative method for predicting cell malignancy and help doctors’ decisions on patient care.

**Abstract:**

Pancreatic cancer (PCA) is one of the most lethal malignancies worldwide with a 5-year survival rate of 9%. Despite the advances in the field, the need for an earlier detection and effective therapies is paramount. PCA high heterogeneity suggests that epigenetic alterations play a key role in tumour development. However, only few epigenetic biomarkers or therapeutic targets have been identified so far. Here we explored the potential of distinct DNA methylation signatures as biomarkers for early detection and prognosis of PCA. PI3K/AKT-related genes differentially expressed in PCA were identified using the Pancreatic Expression Database (*n* = 153). Methylation data from PCA patients was obtained from The Cancer Genome Atlas (*n* = 183), crossed with clinical data to evaluate the biomarker potential of the epigenetic signatures identified and validated in independent cohorts. The majority of selected genes presented higher expression and hypomethylation in tumour tissue. The methylation signatures of specific genes in the PI3K/AKT pathway could distinguish normal from malignant tissue at initial disease stages with AUC > 0.8, revealing their potential as PCA diagnostic tools. *ITGA4*, *SFN*, *ITGA2*, and *PIK3R1* methylation levels could be independent prognostic indicators of patients’ survival. Methylation status of *SFN* and *PIK3R1* were also associated with disease recurrence. Our study reveals that the methylation levels of PIK3/AKT genes involved in PCA could be used to diagnose and predict patients’ clinical outcome with high sensitivity and specificity. These results provide new evidence of the potential of epigenetic alterations as biomarkers for disease screening and management and highlight possible therapeutic targets.

## 1. Introduction

Pancreatic cancer (PCA) remains one of the most mortal malignancies worldwide with a 5-year survival rate of 9%, the lowest of all cancers [1]. This unsettling prognosis is the result of a late diagnosis, due to unspecific early symptoms, lack of useful diagnostic tools, and low efficiency of the therapies currently employed in the clinic [2,3,4]. The only FDA approved biomarker for PCA is the Cancer-Antigen 19-9 with a sensitivity of 60–70% and a specificity of 70–85%. However, CA19-9 is not useful as a diagnostic tool and is only used as prognostic tool to monitor patients’ response to treatment [5]. Once diagnosed, only about 15–20% of the patients are eligible for surgery, which remains the principal therapeutic strategy for PCA followed by chemotherapy and/or radiotherapy according to the distinct disease subtypes [2,6,7]. Despite efforts to develop new diagnostic and therapeutic approaches for PCA, none have reached the clinic and the mortality rates of PCA remain high and with a tendency to increase [1,2]. These facts clearly evidence the importance to further uncover the multiple levels of complexity of this disease.

Cancer results from the acquisition of specific capabilities by cancer cells that allow them to escape the regulatory mechanisms responsible for maintaining cell homeostasis. Cumulative genetic and epigenetic events seem to contribute to this transformation and further differentiate specific cancer types and subtypes. While the genetic landscape of PCA has been known for years [8,9,10,11,12,13], recent efforts have contributed to characterising the epigenetic one [14,15,16,17,18]. The most important genetic events in pancreatic ductal adenocarcinoma (PDAC), the most common type of PCA, are the activation of the oncogene KRAS and the inactivation of the tumour suppressor genes cyclin dependent kinase inhibitor 2A (CDKN2A), tumour protein p53 (TP53), and SMAD4 [8,9,10,11,12,13]. Several lesions such as intraductal papillary-mucinous neoplasm (IPMN), pancreatic intraepithelial neoplasia (PanIN), and mucinous cystic neoplasm can progress and originate PDACs [8]. The precursor lesions mostly associated with tumour development are PanINs which consist of epithelial neoplasms that occur in pancreatic ducts [8]. One of the cellular processes implicated in PCA is the process of acinar to ductal metaplasia (ADM). ADM is a cellular mechanism required for pancreatic tissue regeneration after inflammation or injuries [19]. These ADM lesions can progress to PanIN lesion and eventually progress to pancreatic ductal adenocarcinoma in response to oncogenic signalling [20]. Alterations in the most important PDAC driver genes are also observed in precursor lesions such as PanIN with activation of the oncogene KRAS as an early event of lesion development, which then leads to inactivation of CDKN2A, characteristic of PanIN2 stage. With the progression of the lesion and the establishment of a PanIN3 lesion, TP53 and SMAD4 are inactivated, which are the most commonly observed genetic alterations in PDAC. This is consistent with the progression of these lesions to a malignant state [8,9,10,11,12,13,21,22]. 

More recently, global genomic analysis revealed that several signalling pathways are frequently altered in PCA development including the phosphatidylinositol 3-kinase (PI3K)/AKT pathway [15,23,24,25]. This pathway participates in essential cellular functions such as cell proliferation and apoptosis and its aberrant activation is known to sustain cancer progression [20,26,27,28,29]. Class IA PI3Ks are heterodimeric lipid kinases composed of a p110 catalytic and a smaller regulatory subunit that contains Src-homology 2 (SH2) domains. At the molecular level, extracellular ligands bind to cell surface receptors leading to the SH2 domain-mediated recruitment of the PI3K enzyme to tyrosine phosphorylated proteins at the plasma membrane [29,30]. Upon activation, PI3K generates phosphatidylinositol-3-phosphate (PIP3) by phosphorylating phosphatidylinositol 4, 5-bisphosphate. PIP3 recruits the serine/threonine kinases PDK1 and AKT through their pleckstrin homology domains to the plasma membrane. Membrane-bound AKT is then phosphorylated at serine 473 and threonine 308, leading to its activation and the phosphorylation of downstream substrates many of which are involved in tumour formation and progression. In PCA, activation of the PI3K/AKT pathway drives ADM and the formation of the desmoplastic reaction that is known to be one of the processes involved in therapeutic resistance [20]. The aberrant activation of PI3K/AKT signalling in PCA seems to be due to both genetic and epigenetic events, although the latter are less understood [31,32].

Epigenetic regulation comprises dynamic events that impact on gene expression and play a major role in the heterogeneity of PCA [14,17,18,33]. In agreement, epigenetic regulation is implicated in the normal development and function of the pancreas and deregulation of these mechanisms can result in the development of pancreatic diseases including PCA [18,23,34,35]. Indeed, methylation of genes involved in cell fate decision of pancreatic cells differs between tumour and healthy cells and correlates with patient survival [35]. Among epigenetic events, DNA methylation is currently arising as a potential diagnostic strategy for PCA [33,36,37]. Genes involved in cell cycle regulation and cell proliferation were shown to be differentially methylated during PCA progression [17,33,38]. The methylation of a specific region of the telomerase reverse transcriptase (TERT) gene could distinguish normal pancreatic tissue from early stages of the disease with higher sensitivity and specificity than CA19-9 [39]. Moreover, epigenetic-based biomarkers have become potential targets for early detection of cancer since emerging innovative technology allows the detection of epigenetic alterations in limited amounts of samples as biopsy samples and plasma from blood [40,41,42,43] and even from cell-free DNA and circulating tumour cells in blood [37,44]. In fact, epigenetic-based biomarkers hold some advantages over genetic and protein-based biomarkers. Particularly, DNA methylation profiling is generally focused on specific CpG sites covering smaller regions contrarily to genetic studies that require mutational profiling using full gene length [40]. Additionally, DNA methylation profiling might contribute to increase sensitivity as generally this epigenetic alteration is observed in a higher percentage of tumours [40], occur early in carcinogenesis, and may be specific for PDAC [17,33]. These studies show the potential of epigenetic-based biomarker analysis for cancer diagnosis which is particularly important for PCA where there are no specific and sensitive serological markers for diagnosis.

Here we show evidences that DNA methylation has a role in the activation of PI3K/AKT signalling in PCA. Importantly, we found that alterations in DNA methylation of PI3K/AKT-related genes have the potential to be novel epigenetic biomarkers for early diagnosis of PCA and are capable of predicting survival of the patients and disease recurrence. Our findings lay the groundwork to develop new biomarkers for Pancreatic Cancer screening and management and thus might change the disappointing survival rates of the patients.

## 2. Materials and Methods

### 2.1. Gene Selection

To identify the PI3K/AKT-related genes differentially expressed in PCA we relied on the data freely available on the Pancreatic Expression Database (PED) [45,46,47]. PED contains data not only derived from PCA patients an cell lines but also from patients with benign disease and cancer precursor lesions such as intraductal papillary mucinous neoplasms (IPMNs), pancreatic intraepithelial neoplasias (PanINs), and mucinous cystic neoplasms [45,46,47]. 

We performed a query using the online web resource available at https://pancreasexpression.org/home/ (accessed on 1 January 2017). We selected the genes related with this pathway by selecting the PI3K/AKT signalling (all comparisons) from the Intracellular and 2nd Messenger signalling pathways list. When comparing tumour (*n* = 96) with normal samples (*n* = 57) we established as including criteria a fold-change equal or bigger than 2 and a *p*-value lower than 0.05 (unpaired *t*-test).

Differential expression between normal and tumour pancreatic samples was also assessed using the publicly available data from the GSE28735 dataset [48,49]. Raw data was downloaded from GEO (https://www.ncbi.nlm.nih.gov/geo/query/acc.cgi?acc=GSE28735 (accessed on 1 June 2018) and RMA normalised using BRB array tools developed by Dr. Richard Simon and the BRB-ArrayTools Development Team. Normalised expression data of 45 paired pancreatic tumour-normal samples were used.

### 2.2. DNA Methylation Analysis

To explore if the differentially expressed genes were regulated by DNA methylation, we assessed this parameter using The Cancer Genome Atlas (TCGA) cohort of pancreatic cancer (PAAD) at http://xena.ucsc.edu/ (accessed on 1 March 2018) [50]. Methylation data for PAAD was available for a maximum of 196 samples. However, for the same samples, information regarding other parameters (e.g., clinical data, mRNA expression) might be missing, thus altering sample size (*n*). Thus, for each analysis the *n* is explicitly stated in the respective figure and figure legend. Moreover, we only analysed primary tumour samples and data from patients with no history of neoadjuvant therapy (*n* = 193), as this parameter could independently influence methylation levels. Normal tissue in PAAD is derived from uninvolved tissue surrounding the pancreas including adipose, omentum, subcutaneous tissue or small intestine. We will henceforth refer to those samples collectively as “normal tissue”. Data processing was conducted according to the TCGA data access policies.

Level 3 methylation data derived from the Illumina Infinium HumanMethylation450K array was analysed for PAAD (normal tissue (*n* = 10) and primary tumour (*n* = 183)). From these, 153 tumour samples were pancreatic ductal adenocarcinomas (PDAC) and 8 corresponded to neuroendocrine tumours. The methylation score (β-value) ranges from unmethylated (0) to completely methylated DNA (1) and CpG sites were considered as differentially methylated whenever the difference between primary tumour methylation and normal tissue methylation was higher than 0.2 (|Δβ| ≥ 0.2) and the *p*-value lower than 0.05. CpGs were annotated according to the manifest file for the Infinium HumanMethylation450 version 1.2 CSV format available at https://support.illumina.com/downloads.html (accessed on 1 January 2017) as following: TSS1500 corresponds to the region 200–1500 bases upstream of the transcription start site; 5′UTR corresponds to the 5′ untranslated region, between the TSS and the ATG start site; gene body corresponds to the region between the ATG and stop codon and the 3′UTR corresponds to the region between the stop codon and poly A signal. The CpGs location, putative binding of transcription factors and histone marks in the regions of interest were investigated using the UCSC genome browser available at http://genome.ucsc.edu/ (accessed on 1 January 2017) (Human GRCh37-hg19 genome annotation) [51]. 

Validation of the methylation results was achieved using two additional independent PDAC cohorts: an array dataset (GSE49149) from a genome-wide methylation study comparing 19 samples of adjacent pancreatic tissue and 155 tumour samples [14,16] and the GSE67205 cohort comparing pancreatic tumour (*n* = 11) and pancreatic tissue (*n* = 5) samples digested with Mspl restriction enzyme, bisulfite converted, and sequenced using Hiseq 2000 (Reduced Representation Bisulfite Sequencing) as previously described [35].

### 2.3. Correlation Analysis

To investigate the relationship between DNA methylation and gene expression we assessed the correlation between these two parameters using the Spearman correlation coefficient. For that, gene expression data (level 3 data, RNA-seq Version 2 Illumina; gene-level transcription estimates, as in log2 (x + 1) transformed RSEM normalised count) from the PAAD was retrieved and mapped to corresponding gene methylation status using the unique TCGA identifier barcodes. Due to the lack of expression data from normal tissue in the TCGA cohort, the DNA methylation/gene expression correlation analysis was performed considering only data from pancreatic tumour tissue samples (*n* = 178). 

### 2.4. Clinical Data Analysis

Histological classification and pathological stage were used to investigate the impact of DNA methylation of the selected genes on disease prognosis by crossing the data from HumanMethylation450K array regarding DNA methylation status with the clinical information for each patient. Patient overall survival (OS) and recurrence-free survival (RFS) were also analysed to determine the clinical significance of the observed epigenetic alterations and their potential as biomarkers. Methylation cut-offs for each probe were established by performing Receiver Operating Characteristic (ROC) Curve analysis considering an area under the ROC curve (AUC) with a minimum value of 0.8 to distinguish between healthy and malignant tissues. Results were validated using the GSE49149 cohort. Only the cut-off values that presented sensitivity and specificity values comparable or higher to the values of the CA19-9, the current biomarker for PCA management, were selected for analysis. The patients with methylation values below and above the cut-off value were defined as lowly methylated and highly methylated, respectively. Results were validated using the GSE67205 cohort and a patient-derived xenografts (PDX) cohort (*n* = 75) with whole-genome DNA methylation analysed using the Illumina Infinium HumanMethylation450 Beadchip. Data analysis was performed as previously described [52]. Methylation data is available through ArrayExpress under accession E-MTAB-5008.

### 2.5. Statistical Analysis

Differences between two groups were evaluated using the unpaired *t*-test for data from a normal distribution, except for GSE28735 (paired *t*-test was performed between paired tumour-normal samples). Otherwise, the two-tailed Mann–Whitney test was applied, with a confidence interval of 95%. To analyse the differences between more than two groups we used one-way ANOVA, followed by Kruskal–Wallis test and Dunn’s Multiple Comparison Test. Correlation analysis was performed using the Spearman correlation coefficient. Kaplan–Meier survival curves and respective log-rank tests were generated with GraphPad Prism5.0 (GraphPad Prism 5 Software, San Diego, CA, USA) while univariate and multivariate cox regression analyses of survival were performed using SPSS Statistics v.19 (IBM, 2010, Chicago, IL, USA). For the PDX cohort, the Kaplan–Meier curves and univariate cox regression analysis were performed using the survival R package, applying the cut-off value to optimise the *p*-value. 

### 2.6. Ethics Statement

The PaCaOmics study was registered at www.clinicaltrials.gov with registration number NCT01692873. PDAC samples were collected from January 2012 to December 2015. The study was approved by the local ethics committee (Comité de protection des personnes Sud Méditerranée I) following patient informed consent. All experimental procedures on animals were approved by the ethical committee for animal experimentation and French Ministry of Higher Education and Research (APAFIS# 9562-2016051914513578). All experimental protocols were carried out in accordance with the Guide for the Care and Use of Laboratory Animals (National Academies Press, Washington, DC, USA, 2011).

## 3. Results

Multiple and cumulative genetic and epigenetic alterations contribute to the initiation, progression, and heterogenicity of PCA. Its late diagnosis is the main cause of the poor survival rate observed among PCA patients. Here we propose to evaluate the potential of DNA methylation as a biomarker for PCA early diagnosis and clinical management. Particularly, we focused on the value of PI3K/AKT related genes’ methylation as PCA putative biomarkers.

In order to evaluate epigenetic alterations in genes involved in the PI3K/AKT pathway in PCA, we performed multi-dimensional analysis of data from different cohorts/datasets. We investigated the PI3K/AKT pathway genes, identified at the PED database, that presented differential expression and methylation levels between malignant and healthy pancreatic tissue. For those, we then evaluated the clinical significance of their methylation status.

### 3.1. PI3K/AKT Related Genes Are Deregulated in Pancreatic Cancer

To identify genes involved in the PI3K/AKT pathway differentially expressed between normal and malignant tissue we relied on the PED database. The Pancreatic Expression Landscape integrated in the PED database is the result of a comprehensive meta-analysis of pancreatic gene expression data extracted from numerous published studies and allows the analysis of differential gene expression considering different samples comparison (e.g., pancreatic adenocarcinoma vs. healthy donor), log Fold-change, *p*-value or cell pathway to identify gene deregulation considering specific biological functions [45,46,47]. Our analysis revealed that 15 genes related with PI3K/AKT pathway were differentially expressed between normal and tumour tissue. From those, 13 genes were upregulated in malignant tissue and the remaining 2 were downregulated (Table 1). Using a similar approach in an independent cohort (GSE28735 dataset) we could validate the differential expression between paired normal-tissue samples for stratifin (*SFN*), integrin Subunit Alpha 2 (*ITGA2*), eukaryotic translation initiation factor 4E binding protein 1 (*EIF4EBP1*), and rac family small GTPase2 (*RAC2*) (Supplementary Materials Table S1). Although not all genes had a significant differential expression, most probably because of the small sample size in this cohort (*n* = 45), 13 out of the 15 genes had a consistent expression pattern with the PED analysis.

Since gene expression is highly regulated through epigenetic mechanisms, we interrogated if the differential expression of the genes identified above could be due to such mechanisms, particularly due to alterations in DNA methylation. Since the PED database does not comprise DNA methylation information, we used the data available at the TCGA for the PAAD cohort (miscellaneous of pancreatic adenocarcinomas) to analyse the methylation status of the previously identified differentially expressed genes. Methylation data is unique for each gene in the sense that different genomic locations have different coverage regarding DNA methylation, and thus different genes are covered by a different number of probes (CpG sites). Our analysis revealed that from the 15 differentially expressed genes, 5 were differentially methylated between normal and malignant tissue resulting in 12 differentially methylated CpG sites in total (Table 2). These probes are located within Integrin Subunit Alpha 4 (*ITGA4*), *SFN*, *ITGA2*, phosphatidylinositol-4,5-Bisphosphate 3-Kinase Catalytic Subunit Delta (*PIK3CD*), and phosphoinositide-3-Kinase Regulatory Subunit 1 (*PIK3R1*). In order to validate these results, we used two independent cohorts of patients. First, we compared the methylation values of 19 samples of adjacent pancreatic tissue and 155 tumour samples from a genome-wide 450K array methylation study in pancreatic ductal adenocarcinoma (GSE49149) [16,53]. Indeed, all the 12 CpG sites presented the same methylation pattern as in the TCGA cohort with significant *p*-values although 2 CpGs had a |Δβ|slightly inferior to 0.2 (Supplementary Materials Table S2). Moreover, the methylation status of a small cohort of 11 PDAC vs. 5 pancreatic samples (GSE67205) [35] assessed through Reduced Representation Bisulfite was also analysed. Notably, and although we could only analyse 6 out of the 12 probes since the other CpGs were not sequenced in these samples, the methylation status of *ITGA4*@cg06952671, *ITGA4*@cg21995919, *ITGA4*@cg25024074, *SFN*@cg07786675, SFN@cg13374701, and *PI3KCD*@cg07805542 was consistent with our previous results (Supplementary Materials Table S2 and Figure S1a–f). Since both validation cohorts contained only PDAC samples, we re-analysed the TCGA data focusing on PDCA only and confirmed that the 12 CpGs maintain similar methylation patterns as for the total of PCA samples (Figure S2).

Thus, the validation analysis with PDAC independent cohorts supports our findings when using the TCGA cohort and confirms the methylation profile of these genes in pancreatic cancer.

**Figure 1:**
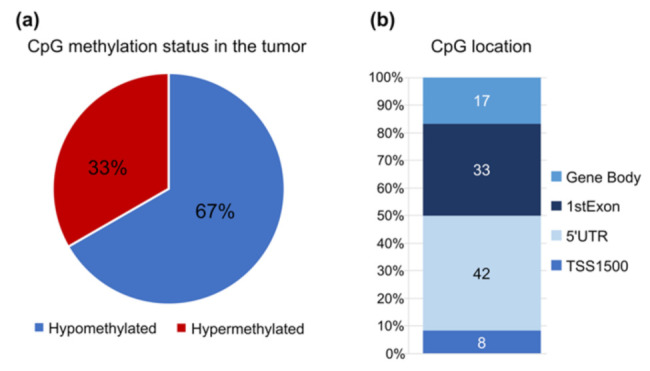
Methylation pattern and genome location of the 12 CpGs in study using the TCGA PCA cohort. (**a**) % of CpGs hypomethylated (blue) and hypermethylated (red) in tumor samples compared to the control. (**b**) Distribution of the 12 probes for the Infinium HumanMethylation450. TSS1500, probes located within 1500 base pairs from the transcription start site (TSS); 5′UTR, 5′ untranslated region.

Since the effect of DNA methylation on gene expression is dependent on the CpGs methylation status and genomic locations within a gene [54], we also investigated these parameters (Table 2 and Figure 1b). From the 12 differentially methylated CpGs located within those 5 genes, 33% of the probes were hypermethylated while 67% were hypomethylated in primary tumour tissue when compared with normal tissue (Figure 1a, Table 2 and Supplementary Materials Table S2). Hypermethylated probes were all located in the *ITGA4* gene.

Regarding their location across the genes, we found that the CpG probes were not evenly distributed along the entire gene region and that approximately 50% of the differentially methylated CpG probes were located in important regulatory regions: the TSS1500 region within 1500 base pairs from the transcription start site and the 5′UTR (Figure 1b). These regions are involved in transcriptional regulation and alteration of their normal methylation patterns can lead to altered gene expression and protein production [54]. Of note, none of the significantly altered CpG sites were at the 3′UTR of the genes.

To uncover if those alterations in DNA methylation could affect gene expression, we performed correlation analysis between DNA methylation and gene expression in matched samples from the TCGA cohort. Due to the small number of normal tissue samples for gene expression (*n* = 4) we used only the primary tumour samples for this analysis. We found that the methylation of the *ITGA4*, *ITGA2*, and *SFN* genes was negatively correlated with gene expression (Table 3). Contrarily, a positive correlation between DNA methylation of *PIK3R1* and *PIK3CD* and their expression was observed (Table 3). Moreover, data from the UCSC Genome Browser shows that the 12 CpGs in study are located in H3K27Ac marks which are frequently found near active regulatory elements thus suggesting that the methylation of these regions has an impact on gene transcription and expression [55,56]. 

To further explore the biomarker potential of the epigenetic alterations here identified, only the genes that presented alterations in the methylation levels and correlated changes in expression levels were selected for the analysis considering the clinical parameters of the patients.

### 3.2. Methylation of PI3K/AKT Related Genes Is Associated with Patients’ Survival

In order to evaluate if the epigenetic alterations here identified could have clinical significance and biomarker potential in PCA, we first performed ROC curve analysis considering the methylation levels of *ITGA4*, *SFN*, *ITGA2*, *PIK3CD*, and *PIK3R1* to evaluate which CpGs could distinguish normal from tumour tissue. In fact, all 12 CpG in analysis presented an AUC > 0.8 evidencing their diagnostic value [57] as promising candidates for PCA detection (Table 2). Additionally, all CpGs presented similar AUC values in the GSE49149 cohort, corroborating our findings (Supplementary Materials Table S2).

**Figure 2:**
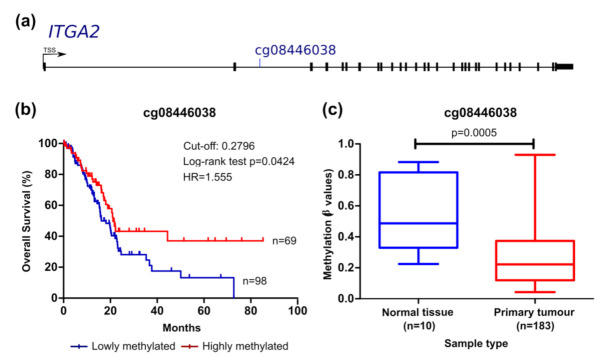
*ITGA2* methylation can predict survival in the TCGA pancreatic cancer cohort. (**a**) Probe location within the gene is shown (scheme is at scale). (**b**) Kaplan–Meier curve for OS (log-rank test). The cut-off value for cg08446038 methylation was 0.2796 with sensitivity and specificity of 58.58% and 87.50%, respectively. Patients with methylation levels inferior and superior to the cut-off were considered as lowly (represented in blue) and highly methylated (represented in red), respectively. (**c**) Comparison of methylation levels between normal and tumour tissue (mean ± SD; Mann–Whitney test). *HR*, hazard ratio.

**Figure 3:**
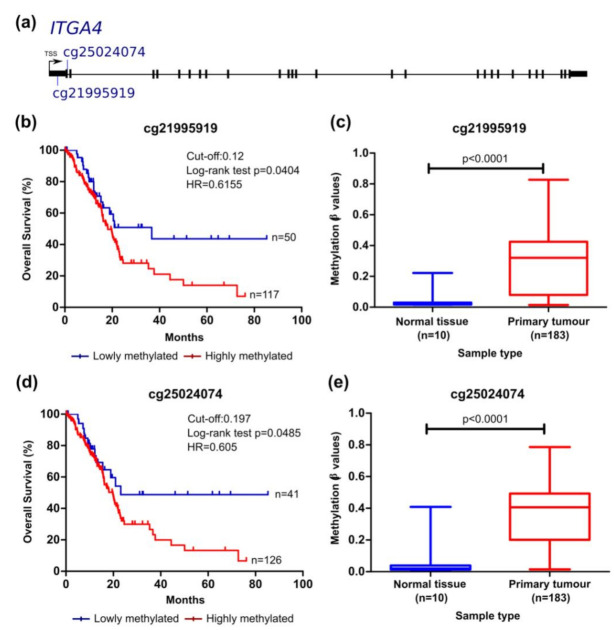
*ITGA4* methylation can predict survival in the TCGA pancreatic cancer cohort. (**a**) Probe location within the gene is shown (scheme is at scale). Kaplan–Meier curve for OS (log-rank test) for (**b**) cg2195919 and (**d**) cg25024074. The cut-off values, sensitivity and specificity values were, respectively, 0.12 (70.41% and 87.50%) and 0.1970 (75.74% and 87.50%). Patients with methylation levels inferior and superior to the cut-off were considered as lowly and highly methylated, respectively. Comparison of methylation levels between normal and tumour tissue (mean ± SD; Mann–Whitney test) for (**c**) cg2195919 and (**e**) cg25024074. *HR*, hazard ratio.

**Figure 4:**
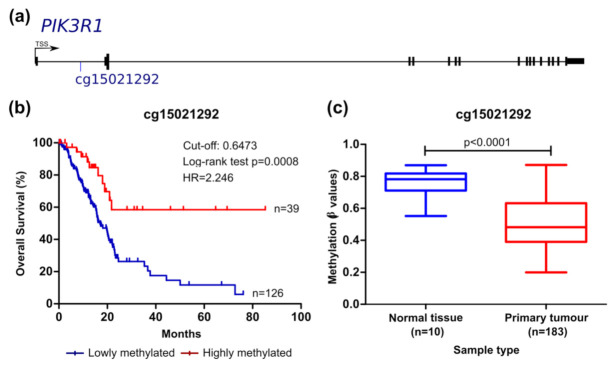
*PIK3R1* methylation can predict survival in the TCGA pancreatic cancer cohort. (**a**) Probe location within the gene is shown (scheme is at scale). (**b**) Kaplan–Meier curve for OS (log-rank test). The cut-off value for cg15021292 was 0.6473, with sensitivity and specificity values of 75.60% and 87.50%, respectively. Patients with methylation levels inferior and superior to the cut-off were considered as lowly and highly methylated, respectively. (**c**) Comparison of methylation levels between normal and tumour tissue (mean ± SD; Mann–Whitney test). *HR*, hazard ratio.

**Figure 5:**
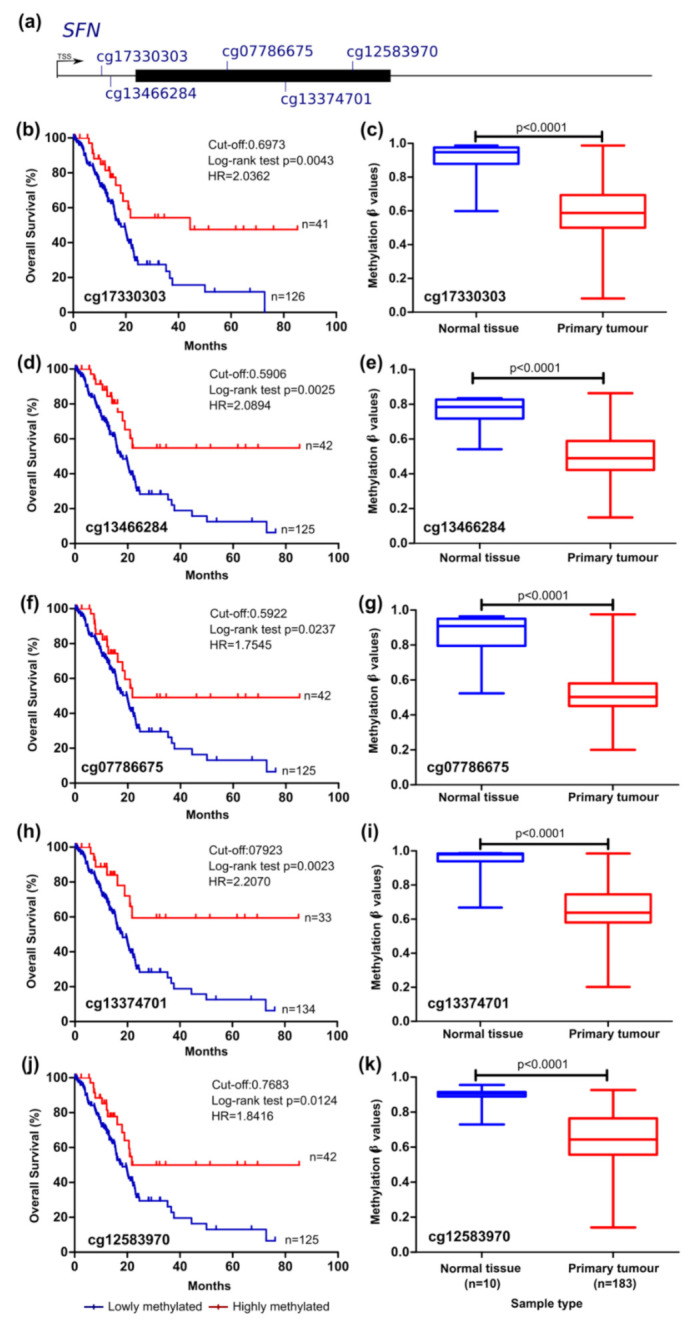
*SFN* methylation can predict survival in the TCGA pancreatic cancer cohort. (**a**) Probe location within the gene is shown (scheme is at scale). Kaplan–Meier curve for OS (log-rank test) (**b**,**d**,**f**,**h**,**j**) and comparison between normal and tumour tissue (mean ± SD; Mann–Whitney test) (**c**,**e**,**g**,**i**,**k**) considering the methylation levels of the probes cg17330303, cg13466284, g07786675, cg13374701, and cg12583970. Cut-off values for Kaplan–Meier curves and sensitivity and specificity values were, respectively, 0.6973 (75.15% and 87.50%); 0.5906 (75.15% and 87.50%); 0.5922 (75.15% and 87.50%); 0.7923 (80.47% and 87.50%); 0.7683 (75.15% and 87.50%). Patients with methylation levels inferior and superior to the cut-off were considered as lowly and highly methylated, respectively. *HR*, hazard ratio.

**Figure 6:**
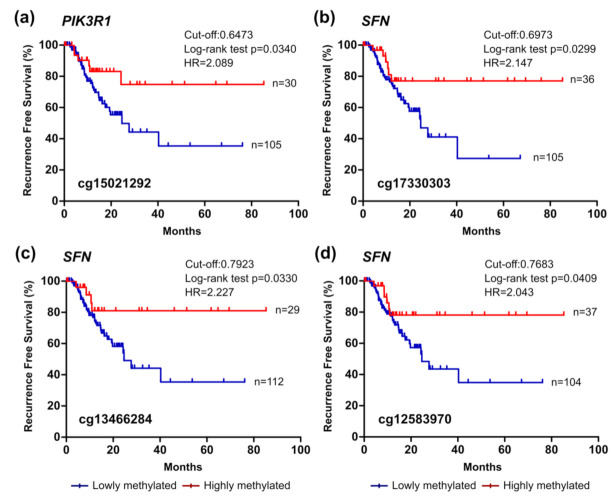
*PIK3R1* and *SFN* methylation can predict recurrence in the TCGA pancreatic cancer cohort. Kaplan–Meier curves for recurrence-free survival (RFS) considering the methylation levels of the probes (**a**) cg15021292 within *PIK3R1* and (**b**–**d**) cg17330303, cg13466284, and cg12583970 within *SFN*. The cut-off values and sensitivity and specificity values were, respectively, 0.6473 (75.60% and 87.50%), 0.6973 (75.15% and 87.50%), 0.7923 (80.47% and 87.50%), and 0.7683 (75.15% and 87.50%). Patients with methylation levels inferior and superior to the cut-off were considered as lowly and highly methylated, respectively. *HR*, hazard ratio.

We then analysed the prognostic value of each CpG considering the overall survival (OS) (Figure 2, Figure 3, Figure 4 and Figure 5) and the recurrence-free survival (RFS) (Figure 6) of patients. Intriguingly, the methylation levels in the *ITGA2*, *ITGA4*, *PIK3R1*, and *SFN* genes were significantly associated with OS and a total of 9 out of the 12 CpG sites differentially methylated were able to predict patient outcome (Figure 2, Figure 3, Figure 4 and Figure 5). Patients with higher methylation levels in *ITGA2*, *PIK3R1*, and *SFN* presented a better prognosis (Figure 2, Figure 4 and Figure 5) while higher levels of *ITGA4* methylation were indicative of a worse prognosis (Figure 3).

The methylation levels of the cg08446038 probe which targets a CpG site located in the body of the *ITGA2* gene was significantly correlated with OS. *ITGA2* encodes for the alpha subunit of an integrin protein involved in cell adhesion [14]. Methylation levels of *ITGA2* at this specific site negatively correlates with gene expression. Patients with lower methylation values, associated with increased gene expression, presented shorter OS (Figure 2, Table 3). 

The methylation levels of the cg21995919 and cg25024074 probes present in the 1st exon of the *ITGA4* gene (5′UTR and coding region, respectively) could also distinguish patients with different survival times (Figure 3). Our analysis revealed that patients with higher methylation values, associated with lower expression of the gene, presented shorter time of survival. This region of the *ITGA4* gene has potential binding sites for both transcriptional activators and repressors according to the UCSC genome browser. Methylation of this region can potentially impair the binding of activators or repressors thus impacting transcription of this gene. 

Considering the epigenetic regulation of the *PIK3R1* gene, which encodes three regulatory isoforms of the PI3K enzyme, lower methylation of the cg15021292 probe significantly correlated with decreased OS of patients with PCA (Figure 4). This probe targets a CpG site located in the TSS1500 and presented a positive correlation with gene expression (Table 2). The CpG site targeted by this methylation probe is integrated in a genomic sequence that can be recognised by proteins involved in both transcription activation and repression (UCSC genome browser). Lower methylation levels of this region were also associated with reduced RFS of patients (Figure 6a).

The analysis of *SFN* epigenetic regulation revealed that the methylation levels of five probes were significantly correlated with the survival of patients: probes cg17330303, cg13466284, cg07786675, cg13374701, and cg12583970. The probes cg17330303 and cg13466284 target CpGs located in the 5′UTR of the gene and the remaining probes target CpGs located in the 1st exon of the gene (Table 2). Methylation of these regulatory regions has been associated with transcriptional repression [54] and, in accordance, here we show that methylation in these CpG sites is negatively correlated with *SFN* expression (Table 3). Additionally, patients with methylation values inferior to the cut-off presented lower time of survival for the five probes (Figure 5). Lower methylation levels of the cg17330303, cg13374701, and cg12583970 probes were also indicative of reduced RFS of the patients (Figure 6b). 

Taken together, these data reveal a strong association of differential methylation of genes related to PI3K/AKT signalling with clinical outcome of patients with PCA. Among five clinical parameters analysed (pathological stage, histological grade, age, gender, and race), grade was the only one that was significantly associated with OS (Supplementary Materials Table S3). Multivariate analyses including grade as the confounding factor showed that ITGA4@cg25024074, ITGA4@cg21995919, PIK3R1@cg15021292, SFN@cg17330303, SFN@cg13466284, SFN@cg13374701 levels were independent predictors of OS (Supplementary Materials Table S4). Moreover, ITGA4@cg06952671 and ITGA4@cg21995919 were also effective in predicting patient outcome in the GSE67205 cohort (Supplementary Materials Figure S1g,h) while ITGA4@cg25024074 and SFN@cg13374701 could predict OS in the PDX cohort (Supplementary Materials Figure S3) thus supporting the potential role of these CpGs as prognostic tools for PCA.

Finally, to understand the biological effect of these epigenetic alterations that could explain the observed differences in patients’ survival, we analysed the methylation of this set of probes considering several clinical and pathological parameters of the patients (e.g., history of chronic pancreatitis and diabetes, primary therapy outcome, histological classification, pathological stage and familiar history of cancer). From the parameters analysed, methylation of selected probes could distinguish between normal tissue and malignant tissue even in early stages (stages I and II) of the disease (Supplementary Materials Figure S4) suggesting it could be a useful diagnostic tool. However, the limited number of samples representative of stage III and stage IV does not allow for drawing meaningful conclusions on methylation changes along the progression of the disease (Supplementary Materials Figure S4).

Furthermore, the methylation of selected CpGs differs between histological subtypes of the disease, when normal, PDAC, and neuroendocrine pancreatic tumour samples are compared (Supplementary Materials Figure S5). While the PDAC methylation pattern is clearly different, the pattern of neuroendocrine pancreatic tumours appear are more similar to the one of normal tissue at the regions analysed, with no statistical differences between the two tissues (Supplementary Materials Figure S5).

Even though the underlying mechanism remains to be established, our data support the development of DNA methylation-based biomarkers for PCA.

## 4. Discussion

The consciousness that early PCA detection is of foremost importance to improve the disappointing survival rates of the patients has increased over the years. Consequently, the implementation of biomarkers for the screening, diagnosis, and clinical management of PCA patients is key to achieve that goal. Here, we explored epigenetic alterations in genes involved in the PI3K/AKT pathway as potential PCA biomarkers. An elevated PI3K/AKT signalling is considered a hallmark of cancer and contributes to the initiation and progression of the disease by promoting cell survival and increasing the ability to migrate and metastasize other tissues [2,20,29,58,59,60]. Due to its central role in cancer, different molecular players of the PI3K/AKT pathway were already reported as potential genetic-based biomarkers or targets for cancer treatment [28,59,61,62,63]. Still, to the best of our knowledge, there are no reports on PI3K/AKT signalling epigenetic-based biomarkers although these present several advantages when compared to genetic or protein-based biomarkers [64]. Particularly, DNA methylation is observed in a higher percentage of tumours which can help increase sensitivity, while its examination is generally focused on specific CpG sites covering a smaller region. DNA methylation signatures are also known to be cell and disease specific while being stable and measurable in different body fluids including blood [33,41,42,44], making this epigenetic mark a promising biomarker for cancer.

In order to evaluate if DNA methylation marks of the PI3K/AKT pathway-related genes are good candidates for PCA biomarkers, we performed a multi-dimensional analysis of data from publicly available datasets. First, we investigated differential expression levels of the PI3K/AKT pathway genes by comparing malignant and healthy pancreatic tissue. With this approach, we identified 15 differentially expressed genes and for these, we then evaluated the clinical significance of their methylation status. 

We proceeded to investigate the methylation levels of those genes taking advantage of the TCGA database which allowed us to correlate DNA methylation levels with gene expression and clinical features of the patients. We found that differential methylation of the *ITGA4*, *SFN*, *ITGA2*, *PIK3CD*, and *PIK3R1* genes allowed the discrimination between normal and tumour tissue evidencing that all 12 CpGs identified were good to excellent diagnostic biomarkers with AUC > 0.8 in two independent cohorts [57]. While the differentially methylated CpG sites in *ITGA4*, *SFN*, and *PIK3R1* are present in regulatory regions of the gene (promoter, 5′UTR, and 1st exon), the CpGs in *ITGA2* and *PIK3CD* are located in the gene body. Still, we could find an association between these methylation marks and gene expression being the majority of CpGs negatively correlated with gene expression. In fact, all CpGs are located in regulatory regions with H3K27Ac marks supporting that these methylation events impact on gene expression [55,56]. DNA methylation of promoter regions has been associated with transcriptional repression whereas gene body methylation is known to occur in genes transcriptionally active [54], but different reports have shown that there are several exceptions to this pattern [39,65]. Additionally, contrarily to that pattern, our results revealed that the methylation of a specific CpG located in the promoter region of the *PIK3R1* gene was positively associated with gene expression while the methylation of a CpG present in the gene body of the *ITGA2* gene was negatively associated with gene expression. It is worth noticing that the association between DNA methylation and gene expression is not linear and that methylation of CpGs can lead either to activation or repression of gene expression by affecting the binding of activator or repressor proteins. Additionally, in this study we only considered as differentially methylated CpG sites that presented a delta beta superior to 0.2 when comparing normal and malignant tissue. Thus, the influence of smaller differences of DNA methylation in gene expression was not assessed. Further studies to infer any type of causality between DNA methylation and gene expression are required and the analysis of the methylation pattern along the entire gene may be more representative of the effect on gene expression rather than specific CpG sites. An additional layer of complexity comes in play when considering the impact of other DNA methylation alterations. The idea that DNA methylation is a more dynamic process than previously believed arose with the possibility of directed DNA methylation and the discovery of enzymes capable of erasing DNA methylation [66,67]. DNA demethylation is the removal or modification of the methyl group from 5-methylcytosine (5mC) [68]. The discovery of the ability of the ten-eleven translocation (TET) enzymes to oxidise 5mC to 5-hydroxymethylcytosine (5hmC) using molecular oxygen as substrate has revolutionised this area and increasing evidence points out to 5hmC as an important gene regulation mark and not just an intermediate in the DNA demethylation pathway: 5hmC is mainly present at genetic regulatory regions, correlates with gene activation, and deregulation of 5hmC patterns is often found in pathologic contexts, including cancer [68,69]. The role of 5hmC in pancreatic cancer is starting to be appreciated. The levels of TET enzymes and the patterns of this epigenetic mark are distinguishable between PDAC and control samples and 5hmC can be found at regulatory regions of PDAC-associated genes such as *MYC*, *KRAS*, *VEGFA*, and *BRD4* promoting gene expression [70,71]. The effect of 5hmC has also been associated with tumour suppressor mechanisms: downregulation of TET1 and 5hmC were associated with transcription of *SFRP2*, which prevents tumour progression trough impairment of WNT pathway, that promotes EMT and cancer progression [71]. Interestingly, it has also been shown that the hydroximethylome of pancreatic cancer patients is altered in cell free DNA samples, potentially paving the way to the development of new diagnostic strategies [72].

Even though the connection between epigenetic regulation, gene expression and patients’ outcomes are far from being completely understood, we investigated the clinical significance of the PI3K/AKT genes methylation status in study. Differential methylation of *ITGA4*, *SFN*, *ITGA2*, and *PIK3R1* revealed to have prognostic value for survival of the patients with higher sensitivity and specificity compared with the currently established biomarker CA19-9 [2]. Moreover, the biomarker potential of ITGA4@cg25024074 and SFN@cg13374701 was validated in a PDX cohort, while ITGA4@cg06952671 and ITGA4@cg21995919 predictive values were corroborated in the GSE67205 cohort, further supporting the prognostic potential of these methylation sites. Notably, the methylation levels of ITGA4@cg25024074, ITGA4@cg21995919, PIK3R1@cg15021292, SFN@cg17330303, SFN@cg13466284, SFN@cg13374701 were independent predictors of OS. Furthermore, the methylation of PIK3R1@cg15021292, SFN@cg17330303, SFN@cg13374701, and SFN@cg12583970 were also associated with the RFS of the patients. Disease recurrence is the main cause of death by the disease and is deeply associated with the resistance to therapy.

Lower methylation levels of the *ITGA2* gene were associated with increased gene expression and worst outcome. Our results are in agreement with a previous study where increased expression of *ITGA2* was correlated with gene hypomethylation and associated with worst prognosis in PCA [14]. Findings published by Chang et al. potentially impacting on disease prognosis also corroborate that *ITGA2* is expressed at a higher level in PCA samples. *ITGA2* encodes an alpha subunit of an integrin protein that can form a heterodimeric protein when associated with a beta subunit [73]. Integrins are cell surface receptors involved in the activation of cellular pathways that play a role in cell motility and survival including the PI3K/AKT pathway [74,75]. Pre-clinical experiments showed that treatment of type I collagen pancreatic cells with anti-integrin α_2_β_1_ antibodies blocking the alpha subunit of integrin prevented their migration capacity. Type I collagen expression is associated with the characteristic desmoplastic reaction of PCA and contributes to treatment resistance and increased cell proliferation. The results of the study from Grzesiak and colleagues reveal α_2_β_1_ as a potential therapeutic target for PCA as it contributes to the malignant phenotype of these groups of cancer cells [76]. Despite its crucial role in cancer, this is the first report highlighting *ITGA2* methylation as potential biomarker for cancer prognosis.

Opposingly, methylation of the *ITGA4* gene that encodes another integrin alpha subunit (α4) is already described as a biomarker for different cancers [77,78,79,80], but not in PCA. Interestingly, we found that higher methylation levels of CpGs present in the first exon of *ITGA4* were associated with lower gene expression and worse outcome. This region represents a potential binding site for both transcriptional activators and repressors. Methylation of this region can potentially impair the binding of activators leading to a decrease in gene expression. Our results corroborate the work of Zhang et al. that described the same effect of DNA methylation in *ITGA4* expression levels and patient’s prognosis [81]. 

One of the major downstream mediators of integrins is PI3K which can be recruited to the plasma membrane upon integrin activation and phosphorylation of focal adhesion kinase (FAK) [75,82]. Phospho-FAK binds to the SH2 domains of the regulatory subunit of PI3K encoded by *PIK3R1*. We found that lower methylation of a CpG site located in the promoter region of the *PIK3R1* gene was associated with reduced gene expression and reduced OS and RFS of the patients. This observation is in line with earlier studies, which showed that the catalytic activity of PI3K is tightly regulated by inhibitory activities of the regulatory subunit [83]. Accordingly, overexpression of *PIK3R1* has been shown to increase the sensitivity of gemcitabine treatment of PCA cells and higher expression of this gene was associated with a decreased in PI3K/AKT signalling pathway activation and increased survival of the patients [84]. Of note, the differential methylation here identified at cg15021292 appears to have independent prognostic value when considering the survival time of the patients. The biomarker potential of *PIK3R1* methylation was also previously reported in esophageal cancer [85].

Additionally, we identified differential expression and epigenetic alterations of a gene that encodes a downstream component related with the PI3K/AKT signalling pathway. *SFN* codes for the 14-3-3 sigma adapter protein involved in the recognition of phosphoserine or phosphothreonine motifs in many signalling proteins. Many of the anti-apoptotic effects of AKT involve 14-3-3 binding to AKT substrates, such as BCL2 associated agonist of cell death (BAD), forkhead box O (FOXO) factors, and Yes1 associated transcriptional regulator (YAP) [29,86]. Here we found that *SFN* hypomethylation was associated with increased gene expression and worse survival of patients with PCA. Accordingly, previous studies have described *SFN* hypomethylation in PCA (reviewed in [17,87]) and identified its increased expression as an independent prognostic PCA biomarker [63]. The clinical significance of *SFN* methylation identified in our analysis is also corroborated by reports in other cancer types where these epigenetic events in *SFN* gene were considered promising biomarkers [88,89]. In addition to predicting OS, our analysis revealed that *SFN* methylation has prognostic value for RFS of the patients, with hypomethylation being indicative of a reduced time without recurrence of the disease. In fact, overexpression of this gene in PCA cell lines was associated with resistance to cisplatin treatment [90]. Resistance to therapeutic agents is one of the major factors that contribute to the discouraging survival rates of the patients. One of the causes of treatment failure is poor diffusion of drugs which is influenced by the formation of a desmoplastic reaction creating a unique and protective microenvironment surrounding cancer cells [20]. This process is impacted by PI3K/AKT signalling pathway which is activated in both pancreatic ductal adenocarcinomas and neuroendocrine pancreatic tumours [91,92]. 

Taken together, our results suggest that the genes here analysed are epigenetically deregulated by aberrant DNA methylation and that these alterations may contribute to PI3K/AKT pathway activation and PCA progression. Thus, our study highlights the potential of these DNA methylation patterns as good candidate biomarkers for PCA. Still, our study derived from deep bio-informatic analyses of published databases and future experimental validation should corroborate our findings. In fact, in silico identification of DNA methylation signatures has proven to be a steppingstone for cancer biomarker identification and further validation [42,43]. Importantly, our results demonstrate the ability to discriminate between groups of patients with different outcomes through the analysis of DNA methylation and to distinguish between normal and malignant tissue even in initial stages of the disease with higher sensitivity and specificity than the currently implemented biomarker revealing its potential as a diagnostic tool for early PCA detection. Advances in the detection of DNA methylation marks in cell-free DNA and circulating tumour cells further support the discovery of promising non-invasive methylation-based biomarkers [37,44].

This study supports the diagnostic and prognostic value of epigenetic alterations in PCA and encourages further studies to complement and validate the observed altered DNA methylation events as PCA biomarkers.

## 5. Conclusions

The implementation of biomarkers for PCA is of foremost importance to counteract the disappointing survival rates of the patients. Profiling of epigenetic alterations holds great potential to improve the molecular evaluation of tumours and help clinicians to adopt the best therapeutic approaches. Differential methylation of the *ITGA4*, *SFN*, *ITGA2*, and *PIK3R1* genes allowed the discrimination between normal and tumour tissue and correlated with patient survival. Using specific cut-offs, we could distinguish between groups of patients regarding their outcome with higher sensitivity and specificity than the currently implemented biomarker for PCA management. This study supports the diagnostic and prognostic value of epigenetic alterations in PCA and encourages further studies to complement the data available at the databases used in this study and to validate the observed alterations as PCA biomarkers.

## Figures and Tables

**Table 1:** PI3K/AKT differentially expressed genes in pancreatic cancer according to the PED database. Fold-change for each probe is based on the comparison between tumour (*n* = 96) and normal tissue samples (*n* = 57) (unpaired *t*-test).

Pathway	Gene	Pathway Activator (↑) or Repressor (↓)	Probe	Fold-Change	*p*-Value	Expression Higher
**PI3K/AKT**	*ITGA4*	↑	205884_at	2.26	<0.0001	*Tumour*
			205885_s_at	2.21	<0.0001	
	*SFN*	↑	209260_at	2.64	0.000154	*Tumour*
			33322_i_at	5.65	<0.0001	
			33323_r_at	5.47	<0.0001	
	*PPP2R5C*	↓	1557718_at	2.47	<0.05	*Tumour*
	*PIK3CD*	↑	203879_at	2.08	<0.001	*Tumour*
	*ITGA2*	↑	205032_at	3.07	<0.0001	*Tumour*
			227314_at	4.77	<0.0001	
	*PIK3R1*	↑	212239_at	1.92	<0.05	*Tumour*
	*AKT3*	↑	212609_s_at	1.9	<0.0001	*Tumour*
			222880_at	1.58	<0.0001	
	*EIF4EBP1*	↑	221539_at	−2.46	<0.0001	*Normal Tissue*
	*INPP5D*	↓	203332_s_at	2.11	<0.001	*Tumour*
	*JAK2*	↓	205842_s_at	2.05	<0.0001	*Tumour*
	*MRAS*	↑	225185_at	1.84	<0.0001	*Tumour*
	*MAP2K2*	↓	213490_s_at	−2.03	<0.0001	*Normal Tissue*
	*MAP3K8*	↑	205027_s_at	3.48	<0.0001	*Tumour*
	*RAC2*	↑	213603_s_at	3.51	<0.0001	*Tumour*
	*PTEN*	↓	1556006_s_at	3.88	<0.05	*Tumour*

**Table 2:** Differentially methylated genes in the TCGA pancreatic cancer cohort. Beta values (β, methylation score) were compared between normal (*n* = 10) and primary tumour (*n* = 183) samples. Patients who received neoadjuvant therapy were excluded from this analysis. CpG sites were considered as differentially methylated whenever the |Δβ| ≥ 0.2 and the *p*-value < 0.05 (Mann–Whitney test). *AUC*, area under the ROC curve; *UTR*, untranslated region; *TSS*, Transcriptional Start Site.

Pathway	Gene	Probe	CpG Location	Mean β Normal Tissue	Mean β Tumour Tissue	|Δβ|	AUC	Methylation Higher	*p*-Value
**PI3K/AKT**	*ITGA4*	cg25652029	5′UTR; 1stExon	0.05121	0.263	0.21179	0.8628	*Tumour*	0.0004
		cg06952671	5′UTR;1stExon	0.02106	0.2828	0.26174	0.9434		<0.0001
		cg21995919	5′UTR; 1stExon	0.04834	0.2738	0.22546	0.8967		0.0003
		cg25024074	1stExon	0.07445	0.3427	0.26825	0.9046		0.0003
	*SFN*	cg17330303	5′UTR; 1stExon	0.8891	0.6121	0.277	0.9087	*Normal Tissue*	0.0002
		cg13466284	5′UTR; 1stExon	0.7461	0.5177	0.2284	0.9183		0.0001
		cg07786675	1stExon	0.8341	0.5408	0.2933	0.9208		0.0001
		cg13374701	1stExon	0.9243	0.677	0.2473	0.9197		0.0001
		cg12583970	1stExon	0.8847	0.6573	0.2274	0.9396		<0.0001
	*PIK3CD*	cg07805542	Gene body	0.6719	0.3175	0.3544	0.8754	*Normal Tissue*	0.0005
	*ITGA2*	cg08446038	Gene body	0.547	0.2715	0.2755	0.8298	*Normal Tissue*	0.0023
	*PIK3R1*	cg15021292	TSS1500	0.7572	0.5152	0.242	0.9063	*Normal Tissue*	0.0002

**Table 3:** Correlation between the 12 CpG methylation levels and gene expression in the TCGA pancreatic cancer cohort. Correlation analysis was performed in tumour samples (*n* = 178) for the 12 differentially methylated CpGs using the two tailed Spearman correlation.

Pathway	Gene Symbol	Probe	Spearman r	*p*-Value	Correlation
**PI3K/AKT**	*ITGA4*	cg25652029	−0.3868	<0.0001	Negative
		cg06952671	−0.3615	<0.0001	Negative
		cg21995919	−0.2992	<0.0001	Negative
		cg25024074	−0.3396	<0.0001	Negative
	*SFN*	cg17330303	−0.5648	<0.0001	Negative
		cg13466284	−0.5833	<0.0001	Negative
		cg07786675	−0.5519	<0.0001	Negative
		cg13374701	−0.6049	<0.0001	Negative
		cg12583970	−0.5921	<0.0001	Negative
	*PIK3CD*	cg07805542	0.2534	0.0011	Positive
	*ITGA2*	cg08446038	−0.4785	<0.0001	Negative
	*PIK3R1*	cg15021292	0.2214	0.0045	Positive

## Data Availability

The data presented in this study are available in supplementary material.

## Supplementary Materials

The following are available online at https://www.mdpi.com/article/10.3390/cancers13246354/s1: Table S1: PI3K/AKT differentially expressed genes in the GSE28735 dataset; Table S2: Differential methylated genes in the GSE49149 and GSE67205 cohorts; Table S3: Cox regression analyses of the clinicopathological parameters and Overall Survival (OS); Table S4: Multivariate Cox regression analyses of the gene methylation and Overall Survival (OS); Figure S1: Methylation status and Overall Survival analysis in pancreatic cancer in the GSE67205 cohort; Figure S2: Methylation status of pancreatic ductal adenocarcinoma samples from the TCGA cohort; Figure S3: *ITGA4* and *SFN* methylation can predict survival in the PDX pancreatic cancer cohort; Figure S4: Methylation of PI3K/AKT related genes discriminates between normal and malignant tissue of different pathological stages in the TCGA cohort; Figure S5: Methylation of the PI3K/AKT related genes differs between distinct histological subtypes of pancreatic cancer in the TCGA cohort.

## References

[1] Siegel R.L., Miller K.D., Jemal A. (2020). Cancer statistics, 2020. CA Cancer J. Clin..

[2] Mizrahi J.D., Surana R., Valle J.W., Shroff R.T. (2020). Pancreatic cancer. Lancet.

[3] Gheorghe G., Bungau S., Ilie M., Behl T., Vesa C.M., Brisc C., Bacalbasa N., Turi V., Costache R.S., Diaconu C.C. (2020). Early Diagnosis of Pancreatic Cancer: The Key for Survival. Diagnostics.

[4] Zhou B., Xu J.W., Cheng Y.G., Gao J.Y., Hu S.Y., Wang L., Zhan H.X. (2017). Early detection of pancreatic cancer: Where are we now and where are we going?. Int. J. Cancer.

[5] Winter J.M., Yeo C.J., Brody J.R. (2013). Diagnostic, prognostic, and predictive biomarkers in pancreatic cancer. J. Surg. Oncol..

[6] Ducreux M., Cuhna A.S., Caramella C., Hollebecque A., Burtin P., Goéré D., Seufferlein T., Haustermans K., Van Laethem J.L., Conroy T. (2015). Cancer of the pancreas: ESMO Clinical Practice Guidelines for diagnosis, treatment and follow-up. Ann. Oncol..

[7] Burns W.R., Edil B.H. (2012). Neuroendocrine Pancreatic Tumors: Guidelines for Management and Update. Curr. Treat. Options Oncol..

[8] Saiki Y., Horii A. (2014). Molecular pathology of pancreatic cancer. Pathol. Int..

[9] Almoguera C., Shibata D., Forrester K., Martin J., Arnheim N., Perucho M. (1988). Most human carcinomas of the exocrine pancreas contain mutant c-K-ras genes. Cell.

[10] Wilentz R.E., Geradts J., Maynard R., Offerhaus G.J., Kang M., Goggins M., Yeo C.J., Kern S.E., Hruban R.H. (1998). Inactivation of the p16 (INK4A) tumor-suppressor gene in pancreatic duct lesions: Loss of intranuclear expression. Cancer Res..

[11] DiGiuseppe J.A., Hruban R.H., Goodman S.N., Polak M., Van den Berg F.M., Allison D.C., Cameron J.L., Offerhaus G.J.A. (1994). Overexpression of p53 protein in adenocarcinoma of the pancreas. Am. J. Clin. Pathol..

[12] Wilentz R.E., Iacobuzio-Donahue C.A., Argani P., McCarthy D.M., Parsons J.L., Yeo C.J., Kern S.E., Hruban R.H. (2000). Loss of expression of Dpc4 in pancreatic intraepithelial neoplasia: Evidence that DPC4 inactivation occurs late in neoplastic progression. Cancer Res..

[13] Hahn S.A., Schutte M., Shamsul Hoque A.T.M., Moskaluk C.A., da Costa L.T., Rozenblum E., Weinstein C.L., Fischer A., Yeo C.J., Hruban R.H. (1996). DPC4, A Candidate Tumor Suppressor Gene at Human Chromosome 18q21.1. Science.

[14] Nones K., Waddell N., Song S., Patch A.-M., Miller D., Johns A., Wu J., Kassahn K.S., Wood D., Bailey P. (2014). Genome-wide DNA methylation patterns in pancreatic ductal adenocarcinoma reveal epigenetic deregulation of SLIT-ROBO, ITGA2 and MET signaling. Int. J. Cancer.

[15] Waddell N., Pajic M., Patch A.-M., Chang D.K., Kassahn K.S., Bailey P., Johns A.L., Miller D., Nones K., Quek K. (2015). Whole genomes redefine the mutational landscape of pancreatic cancer. Nature.

[16] Bailey P., Chang D.K., Nones K., Johns A.L., Patch A.-M., Gingras M.-C., Miller D.K., Christ A.N., Bruxner T.J.C., Quinn M.C. (2016). Genomic analyses identify molecular subtypes of pancreatic cancer. Nature.

[17] Bararia A., Dey S., Gulati S., Ghatak S., Ghosh S., Banerjee S., Sikdar N. (2020). Differential methylation landscape of pancreatic ductal adenocarcinoma and its precancerous lesions. Hepatobiliary Pancreat. Dis. Int..

[18] Lomberk G., Blum Y., Nicolle R., Nair A., Gaonkar K.S., Marisa L., Mathison A., Sun Z., Yan H., Elarouci N. (2018). Distinct epigenetic landscapes underlie the pathobiology of pancreatic cancer subtypes. Nat. Commun..

[19] Storz P. (2017). Acinar cell plasticity and development of pancreatic ductal adenocarcinoma. Nat. Publ. Gr..

[20] Baer R., Cintas C., Therville N., Guillermet-Guibert J. (2015). Implication of PI3K/Akt pathway in pancreatic cancer: When PI3K isoforms matter?. Adv. Biol. Regul..

[21] Guo J., Xie K., Zheng S. (2016). Molecular biomarkers of pancreatic intraepithelial neoplasia and their implications in early diagnosis and therapeutic intervention of pancreatic cancer. Int. J. Biol. Sci..

[22] Hruban R.H., Goggins M., Parsons J., Kern S.E. (2000). Progression model for pancreatic cancer. Clin. Cancer Res. Off. J. Am. Assoc. Cancer Res..

[23] Raphael B.J., Hruban R.H., Aguirre A.J., Moffitt R.A., Yeh J.J., Stewart C., Robertson A.G., Cherniack A.D., Gupta M., Getz G. (2017). Integrated Genomic Characterization of Pancreatic Ductal Adenocarcinoma. Cancer Cell.

[24] Collisson E.A., Bailey P., Chang D.K., Biankin A.V. (2019). Molecular subtypes of pancreatic cancer. Nat. Rev. Gastroenterol. Hepatol..

[25] Wong H.H., Lemoine N.R. (2009). Pancreatic cancer: Molecular pathogenesis and new therapeutic targets. Nat. Rev. Gastroenterol. Hepatol..

[26] Luo J., Manning B.D., Cantley L.C. (2003). Targeting the PI3K-Akt pathway in human cancer: Rationale and promise. Cancer Cell.

[27] Hennessy B.T., Smith D.L., Ram P.T., Lu Y., Mills G.B. (2005). Exploiting the PI3K/AKT pathway for cancer drug discovery. Nat. Rev. Drug Discov..

[28] Conway J.R., Herrmann D., Evans T.J., Morton J.P., Timpson P. (2019). Combating pancreatic cancer with PI3K pathway inhibitors in the era of personalised medicine. Gut.

[29] Fruman D.A., Chiu H., Hopkins B.D., Bagrodia S., Cantley L.C., Abraham R.T. (2017). The PI3K Pathway in Human Disease. Cell.

[30] Hemmings B.A., Restuccia D.F. (2012). PI3K-PKB/Akt Pathway. Cold Spring Harb. Perspect. Biol..

[31] Cheung M., Testa R.J. (2013). Diverse Mechanisms of AKT Pathway Activation in Human Malignancy. Curr. Cancer Drug Targets.

[32] Spangle J.M., Roberts T.M., Zhao J.J. (2017). The emerging role of PI3K/AKT-mediated epigenetic regulation in cancer. Biochim. Biophys. Acta-Rev. Cancer.

[33] Natale F., Vivo M., Falco G., Angrisano T. (2019). Deciphering DNA methylation signatures of pancreatic cancer and pancreatitis. Clin. Epigenet..

[34] Quilichini E., Haumaitre C. (2015). Implication of epigenetics in pancreas development and disease. Best Pract. Res. Clin. Endocrinol. Metab..

[35] Thompson M.J., Rubbi L., Dawson D.W., Donahue T.R., Pellegrini M. (2015). Pancreatic Cancer Patient Survival Correlates with DNA Methylation of Pancreas Development Genes. PLoS ONE.

[36] Syren P., Andersson R., Bauden M., Ansari D. (2017). Epigenetic alterations as biomarkers in pancreatic ductal adenocarcinoma. Scand. J. Gastroenterol..

[37] Brancaccio M., Natale F., Falco G., Angrisano T. (2019). Cell-Free DNA Methylation: The New Frontiers of Pancreatic Cancer Biomarkers’ Discovery. Genes.

[38] Neureiter D., Jäger T., Ocker M., Kiesslich T. (2014). Epigenetics and pancreatic cancer: Pathophysiology and novel treatment aspects. World J. Gastroenterol..

[39] Faleiro I., Apolónio J.D., Price A.J., De Mello R.A., Roberto V.P., Tabori U., Castelo-Branco P. (2017). The TERT hypermethylated oncologic region predicts recurrence and survival in pancreatic cancer. Futur. Oncol..

[40] Heyn H., Esteller M. (2012). DNA methylation profiling in the clinic: Applications and challenges. Nat. Rev. Genet..

[41] García-Giménez J.L., Sanchis-Gomar F., Lippi G., Mena S., Ivars D., Gomez-Cabrera M.C., Viña J., Pallardó F.V. (2012). Epigenetic biomarkers: A new perspective in laboratory diagnostics. Clin. Chim. Acta.

[42] Vrba L., Oshiro M.M., Kim S.S., Garland L.L., Placencia C., Mahadevan D., Nelson M.A., Futscher B.W. (2020). DNA methylation biomarkers discovered in silico detect cancer in liquid biopsies from non-small cell lung cancer patients. Epigenetics.

[43] Nian J., Sun X., Ming S.Y., Yan C., Ma Y., Feng Y., Yang L., Yu M., Zhang G., Wang X. (2017). Diagnostic accuracy of methylated SEPT9 for blood-based colorectal cancer detection: A systematic review and meta-analysis. Clin. Transl. Gastroenterol..

[44] Gall T.M.H., Belete S., Khanderia E., Frampton A.E., Jiao L.R. (2019). Circulating Tumor Cells and Cell-Free DNA in Pancreatic Ductal Adenocarcinoma. Am. J. Pathol..

[45] Chelala C., Hahn S.A., Whiteman H.J., Barry S., Hariharan D., Radon T.P., Lemoine N.R., Crnogorac-Jurcevic T. (2007). Pancreatic Expression database: A generic model for the organization, integration and mining of complex cancer datasets. BMC Genom..

[46] Marzec J., Dayem Ullah A.Z., Pirrò S., Gadaleta E., Crnogorac-Jurcevic T., Lemoine N.R., Kocher H.M., Chelala C. (2017). The Pancreatic Expression Database: 2018 update. Nucleic Acids Res..

[47] Dayem Ullah A.Z., Cutts R.J., Ghetia M., Gadaleta E., Hahn S.A., Crnogorac-Jurcevic T., Lemoine N.R., Chelala C. (2014). The pancreatic expression database: Recent extensions and updates. Nucleic Acids Res..

[48] Zhang G., Schetter A., He P., Funamizu N., Gaedcke J., Ghadimi B.M., Ried T., Hassan R., Yfantis H.G., Lee D.H. (2012). DPEP1 Inhibits Tumor Cell Invasiveness, Enhances Chemosensitivity and Predicts Clinical Outcome in Pancreatic Ductal Adenocarcinoma. PLoS ONE.

[49] Zhang G., He P., Tan H., Budhu A., Gaedcke J., Ghadimi B.M., Ried T., Yfantis H.G., Lee D.H., Maitra A. (2013). Integration of Metabolomics and Transcriptomics Revealed a Fatty Acid Network Exerting Growth Inhibitory Effects in Human Pancreatic Cancer. Clin. Cancer Res..

[50] Goldman M.J., Craft B., Hastie M., Repečka K., McDade F., Kamath A., Banerjee A., Luo Y., Rogers D., Brooks A.N. (2020). Visualizing and interpreting cancer genomics data via the Xena platform. Nat. Biotechnol..

[51] Kent W.J., Sugnet C.W., Furey T.S., Roskin K.M., Pringle T.H., Zahler A.M., Haussler D. (2002). The Human Genome Browser at UCSC. Genome Res..

[52] Nicolle R., Blum Y., Marisa L., Loncle C., Gayet O., Moutardier V., Turrini O., Giovannini M., Bian B., Bigonnet M. (2017). Pancreatic Adenocarcinoma Therapeutic Targets Revealed by Tumor-Stroma Cross-Talk Analyses in Patient-Derived Xenografts. Cell Rep..

[53] Stratford J.K., Bentrem D.J., Anderson J.M., Fan C., Volmar K.A., Marron J.S., Routh E.D., Caskey L.S., Samuel J.C., Der C.J. (2010). A Six-Gene Signature Predicts Survival of Patients with Localized Pancreatic Ductal Adenocarcinoma. PLoS Med..

[54] Jones P.A. (2012). Functions of DNA methylation: Islands, start sites, gene bodies and beyond. Nat. Rev. Genet..

[55] Calo E., Wysocka J. (2013). Modification of Enhancer Chromatin: What, How, and Why?. Mol. Cell.

[56] Creyghton M.P., Cheng A.W., Welstead G.G., Kooistra T., Carey B.W., Steine E.J., Hanna J., Lodato M.A., Frampton G.M., Sharp P.A. (2010). Histone H3K27ac separates active from poised enhancers and predicts developmental state. Proc. Natl. Acad. Sci. USA.

[57] Greiner M., Pfeiffer D., Smith R. (2000). Principles and practical application of the receiver-operating characteristic analysis for diagnostic tests. Prev. Vet. Med..

[58] Hanahan D., Weinberg R.A. (2011). Hallmarks of cancer: The next generation. Cell.

[59] Noorolyai S., Shajari N., Baghbani E., Sadreddini S., Baradaran B. (2019). The relation between PI3K/AKT signalling pathway and cancer. Gene.

[60] Xu W., Yang Z., Lu N. (2015). A new role for the PI3K/Akt signaling pathway in the epithelial-mesenchymal transition. Cell Adh. Migr..

[61] Sirivatanauksorn V., Dumronggittigule W., Dulnee B., Srisawat C., Sirivatanauksorn Y., Pongpaibul A., Masaratana P., Somboonyosdech C., Sripinitchai S., Kositamongkol P. (2020). Role of stratifin (14-3-3 sigma) in adenocarcinoma of gallbladder: A novel prognostic biomarker. Surg. Oncol..

[62] Keizer R.J., Funahashi Y., Semba T., Wanders J., Beijnen J.H., Schellens J.H.M., Huitema A.D.R. (2011). Evaluation of α2-integrin expression as a biomarker for tumor growth inhibition for the investigational integrin inhibitor E7820 in preclinical and clinical studies. AAPS J..

[63] Robin F., Angenard G., Cano L., Courtin-Tanguy L., Gaignard E., Khene Z.E., Bergeat D., Clément B., Boudjema K., Coulouarn C. (2020). Molecular profiling of stroma highlights stratifin as a novel biomarker of poor prognosis in pancreatic ductal adenocarcinoma. Br. J. Cancer.

[64] García-Giménez J.L., Seco-Cervera M., Tollefsbol T.O., Romá-Mateo C., Peiró-Chova L., Lapunzina P., Pallardó F.V. (2017). Epigenetic biomarkers: Current strategies and future challenges for their use in the clinical laboratory. Crit. Rev. Clin. Lab. Sci..

[65] Arechederra M., Daian F., Yim A., Bazai S.K., Richelme S., Dono R., Saurin A.J., Habermann B.H., Maina F. (2018). Hypermethylation of gene body CpG islands predicts high dosage of functional oncogenes in liver cancer. Nat. Commun..

[66] Baylin S.B., Jones P.A. (2011). A decade of exploring the cancer epigenome-biological and translational implications. Nat. Rev. Cancer.

[67] Serra R.W., Fang M., Park S.M., Hutchinson L., Green M.R. (2014). A KRAS-directed transcriptional silencing pathway that mediates the CpG island methylator phenotype. Elife.

[68] Kohli R.M., Zhang Y. (2013). TET enzymes, TDG and the dynamics of DNA demethylation. Nature.

[69] López V., Fernández A.F., Fraga M.F. (2017). The role of 5-hydroxymethylcytosine in development, aging and age-related diseases. Ageing Res. Rev..

[70] Bhattacharyya S., Pradhan K., Campbell N., Mazdo J., Vasantkumar A., Maqbool S., Bhagat T.D., Gupta S., Suzuki M., Yu Y. (2017). Altered hydroxymethylation is seen at regulatory regions in pancreatic cancer and regulates oncogenic pathways. Genome Res..

[71] Wu J., Li H., Shi M., Zhu Y., Ma Y., Zhong Y., Xiong C., Chen H., Peng C. (2019). TET1-mediated DNA hydroxymethylation activates inhibitors of the Wnt/β-catenin signaling pathway to suppress EMT in pancreatic tumor cells. J. Exp. Clin. Cancer Res..

[72] Song C.-X., Yin S., Ma L., Wheeler A., Chen Y., Zhang Y., Liu B., Xiong J., Zhang W., Hu J. (2017). 5-Hydroxymethylcytosine signatures in cell-free DNA provide information about tumor types and stages. Cell Res..

[73] Chang X., Yang M.F., Fan W., Wang L.S., Yao J., Li Z.S., Li D.F. (2020). Bioinformatic Analysis Suggests That Three Hub Genes May Be a Vital Prognostic Biomarker in Pancreatic Ductal Adenocarcinoma. J. Comput. Biol..

[74] Desgrosellier J.S., Cheresh D.A. (2010). Integrins in cancer: Biological implications and therapeutic opportunities. Nat. Rev. Cancer.

[75] Hamidi H., Ivaska J. (2018). Every step of the way: Integrins in cancer progression and metastasis. Nat. Rev. Cancer.

[76] Grzesiak J.J., Bouvet M. (2006). The α2β1 integrin mediates the malignant phenotype on type I collagen in pancreatic cancer cell lines. Br. J. Cancer.

[77] Strelnikov V.V., Kuznetsova E.B., Tanas A.S., Rudenko V.V., Kalinkin A.I., Poddubskaya E.V., Kekeeva T.V., Chesnokova G.G., Trotsenko I.D., Larin S.S. (2021). Abnormal promoter DNA hypermethylation of the integrin, nidogen, and dystroglycan genes in breast cancer. Sci. Rep..

[78] Attia H.R.M., Ibrahim M.H., El-Aziz S.H.A., Hassan N.M., Osman R.A., Hagag H.A., Yassa M.E., Abdelrahman A.H., Salama I.I., Sobeih M.E. (2020). ITGA4 gene methylation status in chronic lymphocytic leukemia. Future Sci. OA.

[79] Zhang X., Wan S., Yu Y., Ruan W., Wang H., Xu L., Wang C., Chen S., Cao T., Peng Q. (2020). Identifying potential DNA methylation markers in early-stage colorectal Cancer. Genomics.

[80] Locke W.J., Guanzon D., Ma C., Liew Y.J., Duesing K.R., Fung K.Y.C., Ross J.P. (2019). DNA Methylation Cancer Biomarkers: Translation to the Clinic. Front. Genet..

[81] Zhang W., Shang S., Yang Y., Lu P., Wang T., Cui X., Tang X. (2020). Identification of DNA methylation-driven genes by integrative analysis of DNA methylation and transcriptome data in pancreatic adenocarcinoma. Exp. Ther. Med..

[82] Cooper J., Giancotti F.G. (2019). Integrin Signaling in Cancer: Mechanotransduction, Stemness, Epithelial Plasticity, and Therapeutic Resistance. Cancer Cell.

[83] Wu H., Yan Y., Backer J.M. (2007). Regulation of class IA PI3Ks. Biochem. Soc. Trans..

[84] Toste P.A., Li L., Kadera B.E., Nguyen A.H., Linh M., Wu N., Madnick D.L., Patel S.G., David W., Donahue T.R. (2016). P85α is a miR target and affects chemosensitivity in pancreatic cancer Paul. J. Surg. Res..

[85] Peng X., Xue H., Lü L., Shi P., Wang J., Wang J. (2017). Accumulated promoter methylation as a potential biomarker for esophageal cancer. Oncotarget.

[86] Dougherty M.K. (2004). Unlocking the code of 14-3-3. J. Cell Sci..

[87] Khan A.A., Liu X., Yan X., Tahir M., Ali S., Huang H. (2021). An overview of genetic mutations and epigenetic signatures in the course of pancreatic cancer progression. Cancer Metastasis Rev..

[88] Husni R.E., Shiba-Ishii A., Nakagawa T., Dai T., Kim Y., Hong J., Sakashita S., Sakamoto N., Sato Y., Noguchi M. (2019). DNA hypomethylation-related overexpression of SFN, GORASP2 and ZYG11A is a novel prognostic biomarker for early stage lung adenocarcinoma. Oncotarget.

[89] Mirza S., Sharma G., Parshad R., Srivastava A., Gupta S.D., Ralhan R. (2010). Clinical significance of Stratifin, ERα and PR promoter methylation in tumor and serum DNA in Indian breast cancer patients. Clin. Biochem..

[90] Neupane D., Korc M. (2008). 14-3-3σ Modulates Pancreatic Cancer Cell Survival and Invasiveness. Clin. Cancer Res..

[91] Eser S., Schnieke A., Schneider G., Saur D. (2014). Oncogenic KRAS signalling in pancreatic cancer. Br. J. Cancer.

[92] Wolin E.M. (2013). PI3K/Akt/mTOR pathway inhibitors in the therapy of pancreatic neuroendocrine tumors. Cancer Lett..

