# Supplementary material for: DNA Methylation of PI3K/AKT Pathway-Related Genes Predicts Outcome in Patients with Pancreatic Cancer: A Comprehensive Bioinformatics-Based Study
Source: Cancers (Basel). 2021 Dec 17;13(24):6354. doi: 10.3390/cancers13246354 (PMC8699150; doi:10.3390/cancers13246354)
Supplement: Supplementary file 1 [file cancers-13-06354-s001.zip › cancers-1491085-supplementary.pdf]

Supplementary Materials

Supplementary Table S1 – PI3K/AKT differentially expressed genes in the GSE28735 dataset. The expression of 15 genes from the PI3K/AKT pathway was compared between tumor and normal samples from the GSE28735 dataset. Raw data was downloaded from GEO (https://www.ncbi.nlm.nih.gov/geo/) and normalized through the Robust Multichip Averaging (RMA) method using BRB array tools. Paired t-test was performed between paired tumor-normal samples (n=45*2) for differential expression analysis.

Pathway	Gene symbol	Probe	Fold-change	*p* value*	Expression higher
**PI3K/Akt**	*ITGA4*	8046695	1.03	0.41	Tumour
	SFN	7899265	1.26	<0.0001	Tumour
	PPP2R5C	7976863	1.00	0.68	Tumour
	PIK3CD	7897482	0.99	0.34	Normal Tissue
	ITGA2	8105267	1.36	<0.0001	Tumour
	PIK3R1	8105778	0.99	0.68	Normal Tissue
	AKT3	7925531	1.01	0.78	Tumour
	EIF4EBP1	8145889	0.92	0.01	Normal Tissue
	INPP5D	8049246	1.04	0.22	Tumour
	JAK2	8154178	1.04	0.18	Tumour
	MRAS	8082965	1.01	0.58	Tumour
	MAP2K2	8032761	1.00	0.88	Normal Tissue
	MAP3K8	7926900	1.03	0.41	Tumour
	RAC2	8075910	1.06	0.01	Tumour
	PTEN	7928959	0.99	0.07	Normal Tissue

* Benjamini hoechberg corrected *p* value

Supplementary Table S2 – Differential methylated genes in the GSE49149 and GSE67205 cohorts. Beta Sheet File from both datasets were downloaded from GEO (https://www.ncbi.nlm.nih.gov/geo/) and beta values (β, methylation score) of the CpGs in study were compared between normal (n=19) and pancreatic tumor (n=155) samples for the GSE49149 cohort and between tumour (n = 11) and pancreatic tissue (n = 5) samples for the GSE67205 cohort**.** *AUC,* Area Under the ROC Curve.

Pathway	Cohort	Gene symbol	Probe	Mean β pancreatic tissue	Mean β tumour tissue	|Δβ|	AUC	Methylation higher	*p* value*
**PI3K/Akt**	**GSE49149**	*ITGA4*	cg25652029	0.115	0.356	0.242	0.908	*Tumour*	*<0.0001**
			cg06952671	0.035	0.387	0.352	0.939		
			cg21995919	0.052	0.388	0.336	0.939		
			cg25024074	0.071	0.427	0.356	0.959		
		SFN	cg17330303	0.800	0.561	0.239	0.894	*Normal Tissue*	*<0.0001**
			cg13466284	0.720	0.530	0.190	0.872		
			cg07786675	0.780	0.511	0.269	0.891		
			cg13374701	0.855	0.621	0.233	0.883		
			cg12583970	0.809	0.633	0.175	0.866		
		PI3KCD	cg07805542	0.609	0.260	0.349	0.937	*Normal Tissue*	*<0.0001**
		ITGA2	cg08446038	0.541	0.269	0.272	0.946	*Normal Tissue*	*<0.0001**
		PIK3R1	cg15021292	0.711	0.506	0.205	0.860	*Normal Tissue*	*<0.0001**
	**GSE67205**	*ITGA4*	cg06952671	0.083	0.309	0.226		*Tumour*	*0.00063*
			cg21995919	0.017	0.187	0.170			*0.0053*
			cg25024074	0.037	0.149	0.112			*0.013*
		SFN	cg07786675	0.896	0.571	0.325		*Pancreatic Tissue*	*0.0054*
			cg13374701	0.890	0.576	0.314			*0.003*
		PI3KCD	cg07805542	0.539	0.155	0.384		*Pancreatic Tissue*	*0.027*

* Bonferoni corrected p value

Supplementary Table S3 –Cox regression analyses of the clinicopathological parameters and Overall Survival (OS). Data was downloaded from the TCGA cohort of pancreatic cancer at http://xena.ucsc.edu/ and patients who received neoadjuvant therapy were excluded (n=183). *Cox proportional hazards regression performed with OS. **Treated as a continuous variable 1: Stage 1, Stage 1A, Stage 1B; 2: Stage 2A, Stage 2B; 3: Stage 3, 4: Stage 4. ***Treated as a continuous variable 1: Well differentiated; 2: Moderately differentiated; 3: Poorly differentiated or undifferentiated. *Nr.*, number; *HR*, Hazard ratio.

Patient Characteristics	Nr.	HR*	*p* value*
**Age**			
Age ≤ 60 (ref.)	57	1.37	0.159
Age > 60	126		
**Pathological Stage****			
Stage 1	1	1.37	0.081
Stage 1A	5		
Stage 1B	15		
Stage 2A	30		
Stage 2B	120		
Stage 3	5		
Stage 4	5		
Unknown	2		
**Gender**			
Male (ref.)	100	1.211	0.342
Female	83		
Grade***			
Well differentiated (ref.)	31	1.466	**0.007**
Moderately differentiated	96		
Poorly differentiated or	53		
Unknown	3		
**Race**			
White (ref.)	160		0.8
Black or African American	7	1.157	0.752
Asian	11	0.769	0.569
Unknown	5		

Supplementary Table S4 – Multivariate Cox regression analyses of the gene methylation and Overall Survival (OS). Data was downloaded from the TCGA cohort of pancreatic cancer at http://xena.ucsc.edu/ and patients who received neoadjuvant therapy were excluded (n=183). *Multivariate cox proportional hazards regression (Backward Wald) performed with OS. *Nr.*, number; *HR*, Hazard ratio. Gene methylation cut-offs were used as described in the methods and main manuscript.

		95% CI for HR		
**TCGA (n=183)**	**HR***	**Lower**	**Upper**	***p* value***
**Step 1**				
Grade	1.413	1.060	1.883	0.019
ITGA2@cg08446038	0.763	0.496	1.173	0.218
**Step 2**				
Grade	1.466	1.112	1.934	0.007
Grade	1.384	1.037	1.846	0.027
ITGA4@cg25024074	1.845	1.037	3.282	0.037
Grade	1.391	1.046	1.849	0.023
ITGA4@cg21995919	1.834	1.092	3.080	0.022
Grade	1.384	1.030	1.860	0.031
PIK3R1@cg15021292	0.481	0.266	0.867	0.015
Grade	1.404	1.051	1.875	0.021
SFN@cg17330303	0.543	0.314	0.941	0.029
Grade	1.337	0.994	1.797	0.054
SFN@cg13466284	0.445	0.245	0.807	0.008
**Step 1**				
Grade	1.397	1.041	1.875	0.026
SFN@cg07786675	0.653	0.371	1.148	0.139
**Step 2**				
Grade	1.466	1.112	1.934	0.007
Grade	1.319	0.977	1.781	0.071
SFN@cg13374701	0.440	0.224	0.865	0.017
Grade	1.350	1.001	1.821	0.049
SFN@cg12583970	0.592	0.333	1.050	0.073

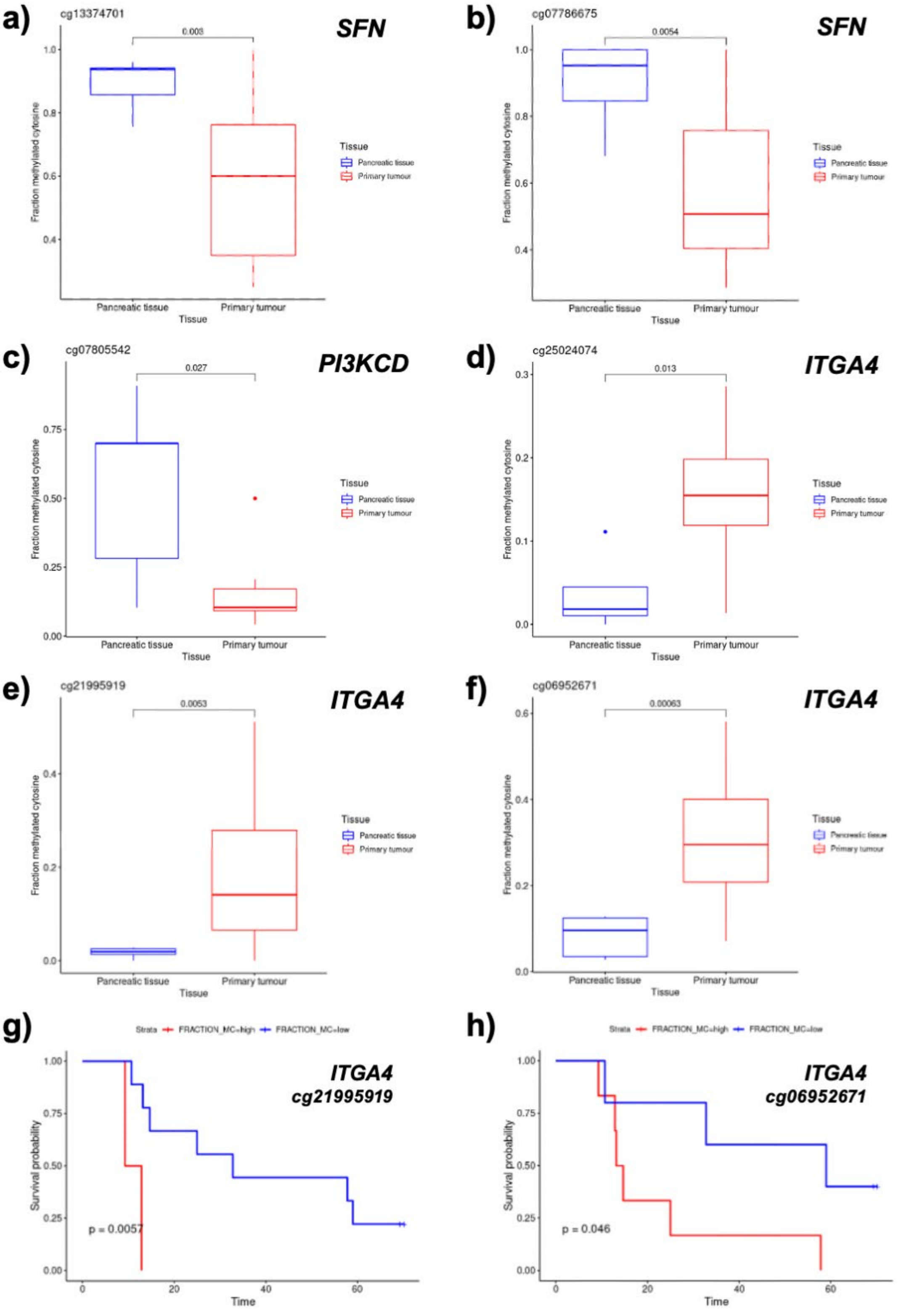

Supplementary Figure S1 – Methylation status and Overall Survival analysis in pancreatic cancer in the GSE67205 cohort. Comparison between pancreatic and tumour tissue of *SFN* (a, b), *PI3KCD* (c) and *ITGA4* (d, e, f). Kaplan-Meier curve for OS considering (g) cg06952671 (cut-off= 0.2632; hazard ratio= 0.21) and (h) cg21995919 methylation (cut-off= 0.2963; hazard ratio= 0.072). Patients with methylation levels inferior and superior to the cut-off were considered as low (represented in blue) and high (represented in red), respectively.

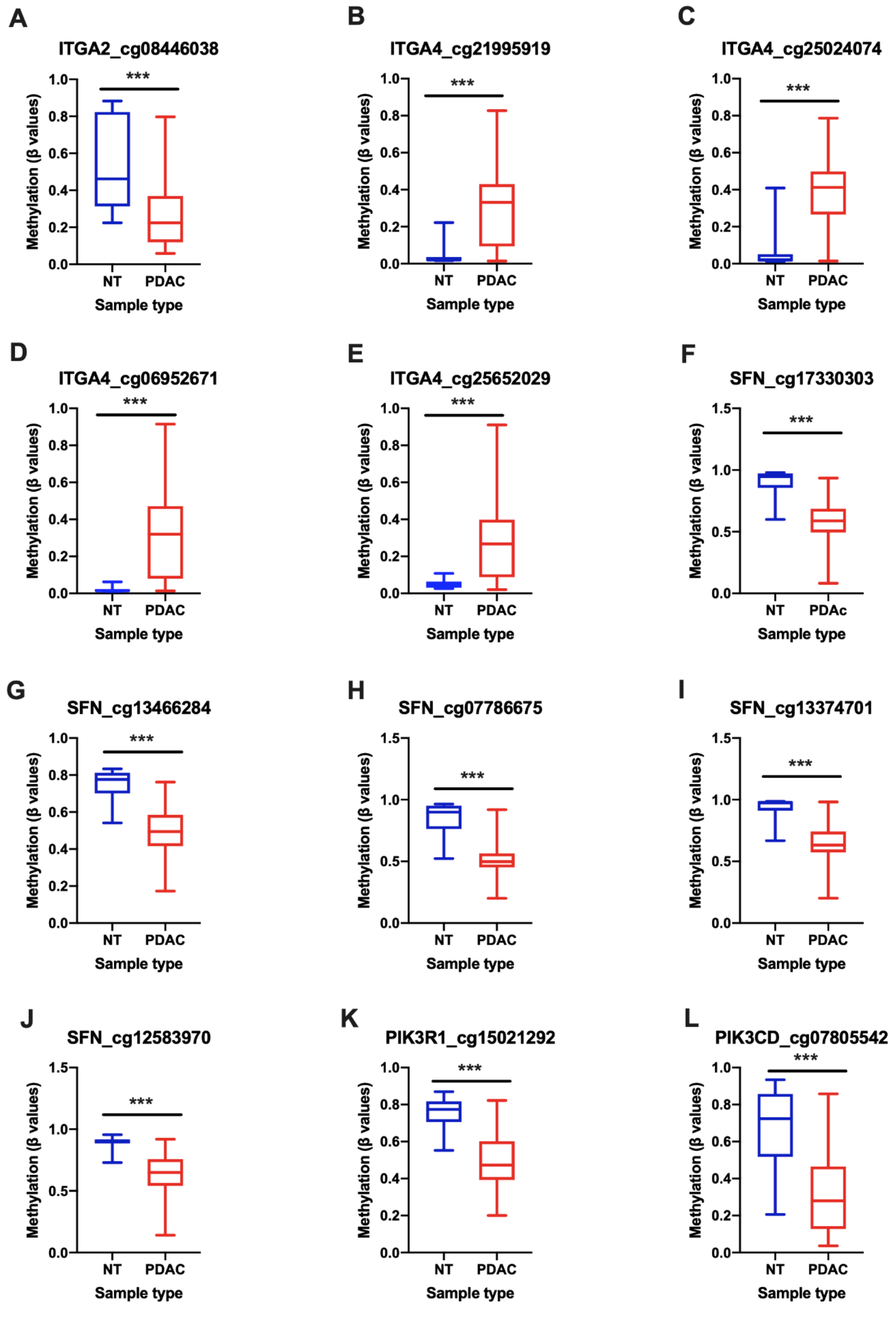

Supplementary Figure S2 – Methylation status of pancreatic ductal adenocarcinoma samples from the TCGA cohort. Beta values (β, methylation score) of the 12 CpGs in study were compared between normal (n = 9) and PDAC tissue (n = 183) using the Mann-Whitney test for differential expression analysis (*** p<0.001). *NT*, normal tissue; *PDAC*, pancreatic ductal adenocarcinoma.

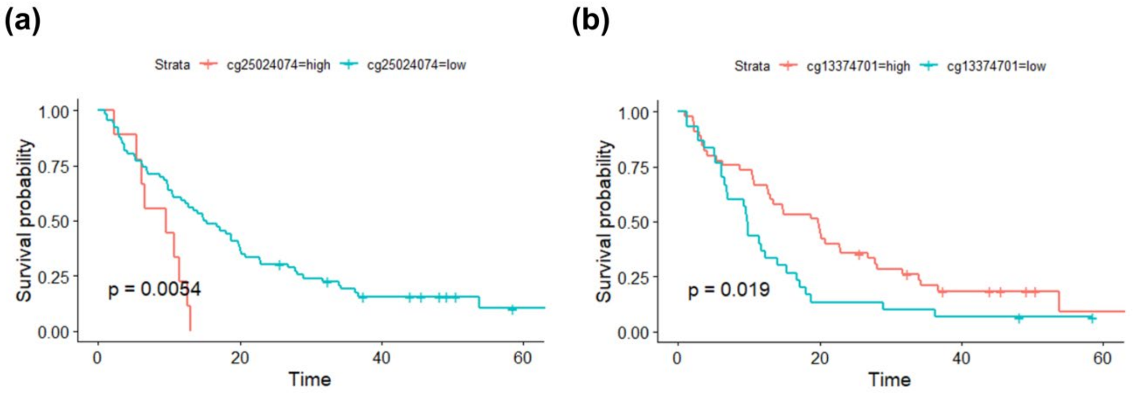

Supplementary Figure S3 – *ITGA4* and *SFN* methylation can predict survival in pancreatic cancer in the PDX cohort. Kaplan-Meier curve for OS considering (a) cg25024074 (cut-off=0.9711; hazard ratio=0.36) and (b) cg13374701 methylation (cut-off=0.3553; hazard ratio=1.18). Patients with methylation levels inferior and superior to the cut-off were considered as low (represented in blue) and high (represented in red), respectively.

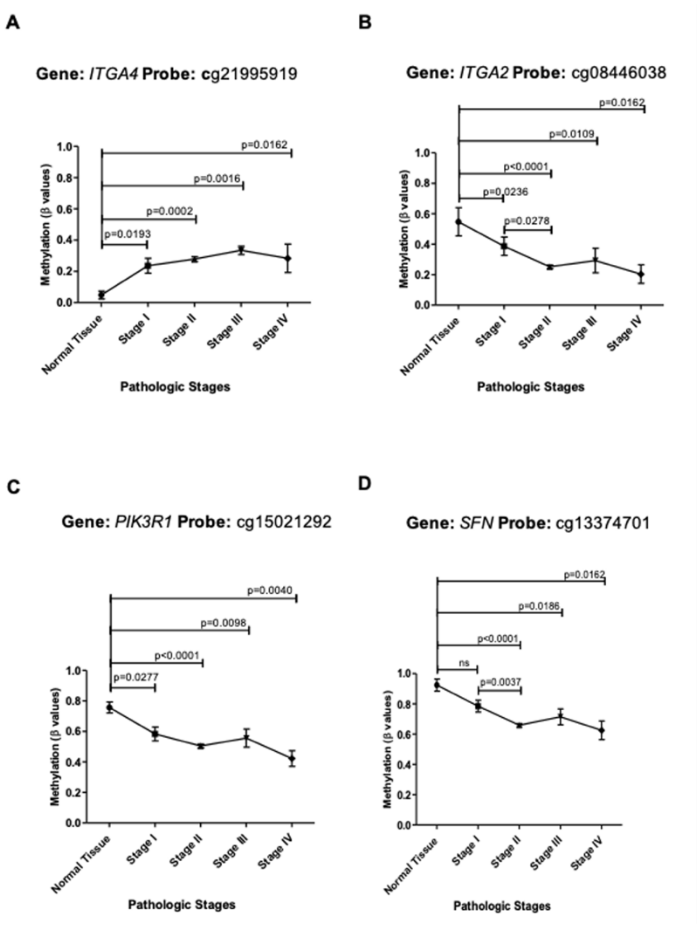

Supplementary Figure S4 - Methylation of PI3K/AKT related genes discriminates between normal and malignant tissue of different pathological stages in the TCGA cohort. Comparison between methylation levels of **(a)** *ITGA4@cg21995919*, **(b)** *ITGA2@cg08446038*, **(c)** *PIK3R1@cg15021292* and **(d)** *SFN@13374701* genes according to the tumour pathological stage (one-way ANOVA, followed by Kruskal-Wallis test and Dunn's Multiple Comparison Test). Number of samples per stage: I (n=21), II (n=150), III (n=5) and IV (n=5). Values are represented as mean ± SEM.

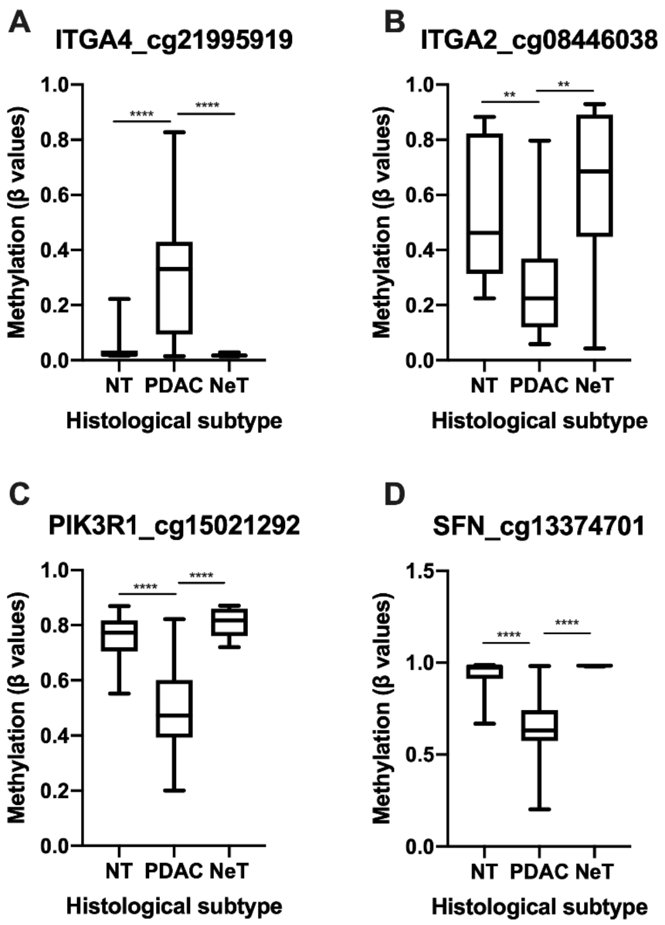

Supplementary Figure S5 - Methylation of the PI3K/AKT related genes differs between distinct histological subtypes of pancreatic cancer in the TCGA cohort. Comparison between DNA methylation of **(a)** *ITGA4@cg21995919*, **(b)** *ITGA2@cg08446038*, **(c)** *PIK3R1@cg15021292* and **(d)** *SFN@cg13374701* considering different histological subtypes of PCA. Statistical analysis of the data was performed using One-way ANOVA followed by Dunn's multiple comparison test (**$p<0.01$; ****$p<0.0001$). *NT*, normal tissue; *PDAC*, pancreatic ductal adenocarcinoma; *NeT*, neuroendocrine tumour.
